# Hidden genetic diversity in snakeskin gourami, *Trichopodus pectoralis* (Perciformes, Osphronemidae), inferred from the mitochondrial DNA *CO1* gene

**DOI:** 10.1080/23802359.2019.1662741

**Published:** 2019-09-12

**Authors:** Min Pau Tan, Thumronk Amornsakun, Mohd Nor Siti Azizah, Ahasan Habib, Yeong Yik Sung, Muhd Danish-Daniel

**Affiliations:** a School of Fisheries and Aquaculture Sciences, Universiti Malaysia Terengganu, Kuala Terengganu, Malaysia;; b Institute of Marine Biotechnology (IMB), Universiti Malaysia Terengganu, Kuala Terengganu, Malaysia;; c Institut Biodiversiti Tropika dan Pembangunan Lestari (BIO-D Tropika), Universiti Malaysia Terengganu, Kuala Terengganu, Malaysia;; d Department of Technology and Industries, Faculty of Science and Technology, Fisheries Technology Program, Prince of Songkla University, Pattani, Thailand;; e Department of Fisheries and Marine Science, Noakhali Science and Technology University, Noakhali, Bangladesh

**Keywords:** *Trichopodus pectoralis*, genetic diversity, mitochondrial DNA, historical translocation, freshwater fish

## Abstract

Eighty-four specimens collected from 13 populations from Malaysia, Thailand, and Vietnam were analysed, revealing 21 putative haplotypes with overall estimated haplotype and nucleotide diversities of 0.79 and 0.0079, respectively. High levels of diversity and an absence of founder effects were observed among populations in peninsular Malaysia. In contrast, populations from Sarawak exhibited low genetic diversity, which is a typical sign of colonies introduced from a single source. Historical translocation of Trichopodus pectoralis from Thailand to Malaysia, as well as to the Philippines, Indonesia, and Myanmar was apparent. Historical introduction of T. pectoralis from Vietnam was also detected in peninsular Malaysia.

## Introduction

The snakeskin gourami, *Trichopodus pectoralis* (Regan 1910) (Perciformes, Osphronemidae), is an important example of an aquaculture candidate in Southeast Asia. This species is widely known among the peoples of Southeast Asian countries (Berra 2011) by different names, i.e. *sepat siam* in Malaysia (Ambak et al. 2010), *pla salid* in Thailand, *cá sac ran* in Vietnam, *trey kawnthor* in Myanmar, and *pa salid* in Laos (Boonsom 1986). Many studies have reported that it is native to the Mekong basin in Laos, Thailand, Cambodia, and Vietnam as well as the Chao Phraya basin (Boonsom 1986; Kottelat 2001) and that it has been widely introduced to Malaysia (Smith 1945; Boonsom 1986), India, Bangladesh, Sri Lanka, Indonesia, and the Philippines (Boonsom 1986). This piscine species is an important ornamental and food fish. Nevertheless, its population size declined due to habitat loss and degradation, especially in Thailand (Vidthayanon 2012).

Understanding the genetic diversity and structure of an economically important species is crucial prior to management planning and the development of a conservation strategy (Sousa-Santos et al. 2016). Such knowledge helps to determine what to conserve and where and improves the understanding of the genetic distribution and phylogenetic relationships of species of interest. Mitochondrial DNA (mtDNA) markers, particularly *cytochrome c oxidase subunit 1* (*CO1*), have been proven to be powerful tools for revealing species identities (Kochzius et al. 2010; Ward 2012), phylogeographic patterns (Tan et al. 2015; Taboada and Pérez-Portela 2016) and the genetic diversity of native and non-native aquatic species (Jamsari et al. 2011; Lejeusne et al. 2015). These markers are potentially useful for revealing contemporary and historical conspecific introductions (Geller et al. 2010; Lejeusne et al. 2011; Tan et al. 2012).

A previous report on the population genetics of *T. pectoralis* from Thailand used isozymes and morphological analysis methods (Prasertwiriyakul and Baoprasertkul 1999). There were high genetic identity coefficients among the surveyed populations (Samutprakan, Pitsanulok, Suphanburi, Ubon Ratchathani, and Pattanee) ranging from 0.923 to 0.985. In another study, the phylogenetic relationships of *T. pectoralis* were inferred based on whole mitochondrial genomes, supporting a greater genetic proximity between *T. pectoralis* from Thailand and Malaysia than between Vietnam and Malaysia or Vietnam and Thailand (Gan et al. 2017); however, the population genetics of this species are still poorly known. Therefore, in this study, the mtDNA *CO1* gene was sequenced to investigate the genetic diversity and population connectivity of *T. pectoralis* collected from Malaysia, Vietnam, and Thailand, with the inclusion of GenBank DNA sequences of *T. pectoralis* from the Philippines, Indonesia, and Myanmar for phylogenetic analysis. The specific aims were to characterize the genetic diversity and define the population structure and phylogenetic relationships of *T. pectoralis* within the habitats where it occurs, with special attention being paid to Malaysian populations.

## Materials and methods

### Sampling, DNA extraction, and PCR amplification

**Figure 1.**
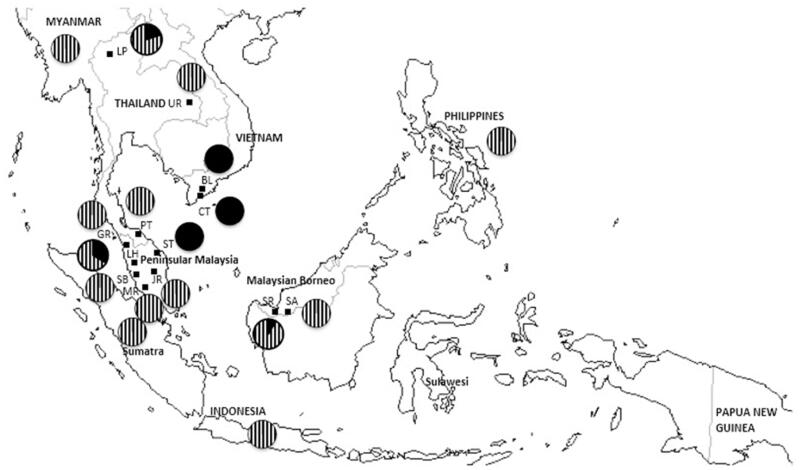
*Trichopodus pectoralis* locality distribution. Samples of Vietnam (solid black) and Thailand (vertical line) assemblage is presented proportionally in pie chart.

Random sampling of 13 wild *T. pectoralis* populations from Malaysia, Vietnam and, Thailand was conducted, and the sampling locations were divided into six regions (Table 1, Table S1, and Figure 1). Live specimens from wild populations were collected from local fishermen, and the catch localities were determined. Small pieces of individual fin rays were cut and preserved in 1.5 ml tubes containing 95% ethanol solution until use. The representative *T. pectoralis* samples collected from Vietnam, Thailand, and Malaysia were stored at the Institute of Oceanography, Universiti Malaysia Terengganu, with voucher ID UMTGen01310-01312.

**Table 1.** Haplotype diversity (H) and nucleotide diversity (*π*) of *T. pectoralis* based on *CO1* sequences. Standard deviation (SD).

Country	Region	Population	*N*	#*V*	*n*H	Genetic diversity			
						Hd	SD	*π*	SD
Malaysia	East Peninsular Malaysia	(1) JR	5	4	3	0.80	0.16	0.0033	0.0026
		(2) ST	5	22	5	1.00	0.13	0.0170	0.0109
	West Peninsular Malaysia	(3) GR	4	3	2	0.60	0.18	0.0029	0.0024
		(4) LH	6	11	3	0.60	0.22	0.0077	0.0050
		(5) SB	2	2	2	1.00	0.50	0.0033	0.0040
	Southern Peninsular Malaysia	(6) MR	4	2	3	0.83	0.22	0.0019	0.0018
	Malaysian Borneo	(7) SA	13	3	3	0.29	0.16	0.0010	0.0009
		(8) SR	12	1	2	0.17	0.13	0.0003	0.0004
	Subtotal	Peninsular Malaysia (east, west, and southern)	**26**	**26**	**11**	**0.86**	**0.05**	**0.0122**	**0.0066**
		Malaysian Borneo	**25**	**3**	**3**	**0.29**	**0.11**	**0.0007**	**0.0007**
		(Malaysia)	**51**	**26**	**12**	**0.66**	**0.07**	**0.0075**	**0.0042**
Vietnam	Vietnam	(9) CT	3	4	3	1.00	0.27	0.0049	0.0043
		(10) BL	3	14	3	1.00	0.27	0.0153	0.0121
	Subtotal	(Vietnam)	**6**	**17**	**6**	**1.00**	**0.10**	**0.0132**	**0.0083**
Thailand	Thailand	(11) LP	5	6	3	0.70	0.22	0.0043	0.0032
		(12) PT	6	3	2	0.33	0.22	0.0016	0.0015
		(13) UR	16	3	3	0.66	0.07	0.0019	0.0015
	Subtotal	(Thailand)	**27**	**8**	**6**	**0.81**	**0.04**	**0.0035**	**0.0023**
	Total		**84**	**28**	**21**	**0.79**	**0.04**	**0.0079**	**0.0043**
	Mean		**6.5**	**6**	**2.8**	**0.69**	**0.29**	**0.0050**	**0.0053**

Sample size (*N*), number of variable sites (#*V*), number of haplotype (*n*H), haplotype diversity (Hd), nucleotide diversity (*π*), standard deviation (SD). JR: Jerantut; ST: Setiu; GR: Guar; LH: Lahat; SB: Sungai Besar; MR: Muar; SA: Seri Aman; SR: Serian; CT: Can Tho; BL: Bac Lieu; LP: Lamphun; PT: Pattani; UR: Ubon Ratchathani. The values are in bold to highlight the subtotal, total and mean values.

Total genomic DNA was isolated from the fin tissue using the AQUAGENOMIC^TM^ kit. PCR amplification was carried out in a volume of 30 µl using 100 ng of genomic DNA, each primer at 0.24 µM, 0.20 mM dNTP, 1 × PCR buffer, 1 mM MgCl_2_, and 0.08 U of *Taq* polymerase (all from iNtRON), in an MJ PTC-200 Thermal Cycler (MJ Research, Waltham, MA). Amplification of the mtDNA *CO1* gene was conducted using the primer pair FishF2 (5″ TCGACTAATCATAAAGATATCGGCAC-3″) and FishR2 (5″-ACTTCAGGGTGACCGAAGAATCAGAA-3″) (Ward et al. 2005). The PCR temperature profile consisted of an initial incubation at 95 °C for 4 min, 35 cycles of 94 °C for 60 s, 60 °C for 60 s, 72 °C for 120 s, a final extension at 72 °C for 20 min, and a final hold at 4 °C. The PCR products were visualized in a 1.7% agarose gel stained with SyBr Safe to confirm successful amplification. All products were sent for DNA sequencing (First BASE Laboratories Sdn Bhd, Selangor, Malaysia) and reading from single DNA strands.

### Data analysis

Multiple sequences were aligned and edited manually using ClustalW implemented in MEGA version 6.0 (https://www.megasoftware.net/) (Tamura et al. 2013). The DNA sequences were translated into amino acids to ensure accurate alignment. All haplotype sequences were deposited in GenBank under accession numbers KX817191-817211. The number of haplotypes, haplotype diversity (Hd) and nucleotide diversity (*π*) were calculated to describe DNA polymorphism at each sampling site using Arlequin version 3.5 (http://cmpg.unibe.ch/software/arlequin35/) (Excoffier and Lischer 2010).

A phylogenetic tree was constructed using the maximum likelihood (ML) method in MEGA 6.0. GenBank sequences of *T. pectoralis* from Indonesia (KU692922-692927, KM213050), the Philippines (HQ682726-682730) and Myanmar (LC190090) were included for spatial comparison, and single sequences from the three-spot gourami, *T. trichopterus* (JN896639), moonlight gourami, *Trichopodus microlepis* (KF805360), and pearl gourami, *Trichopodus leeri* (KR029983), were included as out-group taxa. The confidence levels at each node were assessed by 1000 bootstrap replications (Hall 2013).

A spatial analysis of molecular variance was conducted using SAMOVA version 2.0 (Dupanloup 2016) to identify genetically similar groups of populations and to evaluate the amount of genetic variation between the partitions. The optimal number of groups (*k*) was determined based on the highest variance between groups (*F*_CT_), incorporating information on haplotype divergence and geographical proximity.

## Results and discussion

The overall haplotype (Hd) and nucleotide diversity (*π*) were 0.79 ± 0.04 (mean = 0.69 ± 0.29) and 0.0079 ± 0.0043 (mean = 0.0050 ± 0.0053), respectively (Table 1), indicating a relatively high genetic diversity of *T. pectoralis* within the studied areas. However, closer inspection according to country indicated that individuals from Vietnam were the most diversified (Hd = 1.00 ± 0.10; *π* = 0.0132 ± 0.0083), followed by those from Thailand (Hd = 0.81 ± 0.04; *π* = 0.0035 ± 0.0023) and Malaysia (Hd = 0.66 ± 0.07; *π* = 0.0075 ± 0.0066) (Table 1). Interestingly, the segregation of Malaysian populations into two geographical areas (i.e. peninsular and Malaysian Borneo) resulted in a much higher diversity estimate for peninsular Malaysia (Hd = 0.86 ± 0.05; *π* = 0.0122 ± 0.0066), which was even higher than that for the Thai samples, while Malaysian Borneo presented the lowest genetic diversity among all examined regions (Hd = 0.29 ± 0.11; *π* = 0.0007 ± 0.0007).

While it was not surprising to observe highly diversified individuals in Vietnam and Thailand as the countries of origin (Boonsom 1986; Kottelat 2001), the identification of a relatively large gene pool in peninsular Malaysia was rather unexpected. It is commonly observed that introduced populations tend to show a founder effect or low diversity, as only part of the DNA composition of the original populations is dispersed (Wares et al. 2005; Excoffier et al. 2009); however, this study revealed a different situation. This result could be a consequence of propagule pressure and multiple introductions promoting genetic admixture, hybridization, and introgression (Lejeusne et al. 2015) reviewed in Roman and Darling (2007). Lejeusne et al. (2015) reported similar observations in the introduced European and northwest Atlantic populations of the oriental shrimp *Palaemon macrodactylus*, which exhibited higher haplotypic diversity than most native populations from Japan (and likely South Korea and China).

The phylogenetic tree inferred *via* the ML method reveals clustering of the 21 haplotypes into two major lineages: a lineage of Thai origin (Clade I) and a lineage of Vietnamese origin (Clade II) (Figure S1). Clade I consists of haplotypes found in all regions (except Vietnam), including the Philippines, Indonesia, and Myanmar, indicating the close kinship of haplotypes across these vast geographical areas. Welcomme (1988) reported that the *T. pectoralis* occurring in Indonesia were introduced from Malaysia; however, it is more likely that the actual origin was Thailand based on the present molecular data, as supported by Boonsom (1986). We postulate that the introduction was human-mediated and not associated with natural dispersal because the species is known to be a non-migratory fish (Vidthayanon 2012), and there were no palaeo river systems traversing both regions (Voris 2000). On the other hand, clade II comprises haplotypes from CT and BL (both populations from Vietnam), ST (eastern peninsular Malaysia), and LH (western peninsular Malaysia), indicating probable historical introduction of the *T. pectoralis* from Vietnam into peninsular Malaysia.

For SAMOVA, *k* = 3 clusters returned the highest between-group variation (*F*_CT_, 74.99%), segregating 1) Vietnam (CT), 2) eastern peninsular Malaysia (ST), and 3) other populations. This result is not in accord with our gene tree, in which only two lineages were detected. When *k* = 2 was prompted, CT and ST were grouped together with *F*_CT_ = 74.73%, indicating close kinship between them. Additionally, increasing the number of *k* resulted in a decreasing value of *F*_CT_. The higher *F*_CT_ value obtained for *k* = 3 was expected because the proportion of *F*_SC_ was reduced due to the smaller number of populations within each group, thus reducing the differences between them (Dupanloup et al. 2002). Furthermore, the *F*_ST_ index is not sensitive to *k* evaluation. Therefore, we propose that the *T. pectoralis* present in Malaysia consisted of two distinct groups with origins in Thailand and Vietnam. This study revealed strong invasive potential of *T. pectoralis* in Malaysia, an important criterion for a cultured species to adapt and reproduce in a newly introduced environment.

## Supplementary Material

Supplemental Material

## References

[CIT0001] Ambak MA, Isa MM, Zakaria MZ, Abd Ghaffar M 2010 Fishes of Malaysia. Terengganu (Malaysia): Universiti Malaysia; p. 334.

[CIT0002] Berra TM 2011 Freshwater fish distribution. Chicago (IL): University of Chicago Press; p. 615.

[CIT0003] Boonsom J 1986 Pla-salid (*Trichogaster pectoralis* Regan). A life history and manual for culture. Thai Fishery Gazette. 39:589–601.

[CIT0004] Dupanloup I 2016 SAMOVA 2.0: A program to define the genetic structure of populations by a simulated annealing approach. [Bern (Switzerland)]: University of Bern; [accessed 2018 Oct 11]. http://cmpg.unibe.ch/software/samova2/.

[CIT0028] Dupanloup I, Schneider S, Excoffier L 2002 A simulated annealing approach to define the genetic structure of populations. Mol Ecol. 11:2571–81. doi: 10.1046/j.1365-294X.2002.01650.x 12453240

[CIT0005] Excoffier L, Foll M, Petit RJ 2009 Genetic consequences of range expansions. Annu Rev Ecol Evol Syst. 40:481–501.

[CIT0006] Excoffier L, Lischer HE 2010 Arlequin suite ver 3.5: a new series of programs to perform population genetics analyses under Linux and Windows. Mol Ecol Resour. 10:564–567. 21565059 10.1111/j.1755-0998.2010.02847.x

[CIT0007] Gan HM, Amornsakun T, Tan MP 2017 The complete mitochondrial genome of the snakeskin gourami, *Trichopodus pectoralis* (Regan 1910) (*Teleostei: Osphronemidae*). Mitochondrial DNA Part B Resources. 2:148–149. 10.1080/23802359.2017.1298418 PMC7800780 33473747

[CIT0008] Geller JB, Darling JA, Carlton JT 2010 Genetic perspectives on marine biological invasions. Ann Rev Mar Sci. 2:367–393. 10.1146/annurev.marine.010908.163745 21141669

[CIT0009] Hall BG 2013 Building phylogenetic trees from molecular data with MEGA. Mol Biol Evol. 30:1229–1235. 23486614 10.1093/molbev/mst012

[CIT0010] Jamsari AFJ, Tan MP, Siti Azizah MN 2011 Genetic structure of the snakeskin murrel, *Channa striata* (*Channidae*) based on the cytochrome c oxidase subunit I gene: Influence of historical and geomoirphological factors. Genet Mol Biol. 34:152–160. 21637559 10.1590/S1415-47572011000100026 PMC3085362

[CIT0011] Kochzius M, Seidel C, Antoniou A, Botla SK, Campo D, Cariani A, Vazquez EG, Hauschild J, Hervet C, Hjörleifsdottir S, et al. 2010 Identifying fishes through DNA barcodes and microarrays. PLoS One. 5:e12620. 20838643 10.1371/journal.pone.0012620 PMC2935389

[CIT0012] Kottelat M 2001 Fishes of Laos. Sri Lanka: WHT Publication Ltd; p. 198.

[CIT0013] Lejeusne C, Bock DG, Therriault TW, MacIsaac HJ, Cristescu ME 2011 Comparative phylogeography of two colonial ascidians reveals contrasting invasion histories in North America. Biol Invasions. 13:635–650.

[CIT0014] Lejeusne C, Saunier A, Petit N, Béguer M, Otani M, Carlton JT, Rico C, Green AJ 2015 High genetic diversity and absence of founder effects in a worldwide aquatic invader. Sci Rep. 4:5808. 10.1038/srep05808 PMC5376162 25060780

[CIT0015] Prasertwiriyakul S, Baoprasertkul P 1999 Population genetic structures of the snakeskin gourami, *Trichogaster pectoralis* (Regan, 1910) in Thailand [1999]. Thailand: Thai National AGRIS Centre. Technical Paper No. 22.

[CIT0016] Roman J, Darling JA 2007 Paradox lost: genetic diversity and the success of aquatic invasions. Trends Ecol Evol. 22:454–464. 17673331 10.1016/j.tree.2007.07.002

[CIT0017] Smith H 1945 The freshwater of Siam, or Thailand. Washington (DC): Government Printing Office; p. 622.

[CIT0018] Sousa-Santos C, Robalo JI, Pereira AM, Branco P, Santos JM, Ferreira MT, Sousa M, Doadrio I 2016 Broad-scale sampling of primary freshwater fish populations reveals the role of intrinsic traits, inter-basin connectivity, drainage area and latitude on shaping contemporary patterns of genetic diversity. PeerJ. 4:e1694. 26966653 10.7717/peerj.1694 PMC4782715

[CIT0019] Taboada S, Pérez-Portela R 2016 Contrasted phylogeographic patterns on mitochondrial DNA of shallow and deep brittle stars across the Atlantic-Mediterranean area. Sci Rep. 6:32425. 27585743 10.1038/srep32425 PMC5009426

[CIT0020] Tamura K, Stecher G, Peterson D, Filipski A, Kumar S 2013 MEGA6: molecular evolutionary genetics analysis version 6.0. Mol Biol Evol. 30:2725–2729. 24132122 10.1093/molbev/mst197 PMC3840312

[CIT0021] Tan M, Jamsari A, Muchlisin Z, Siti Azizah MN 2015 Mitochondrial genetic variation and population structure of the striped snakehead, *Channa striata* in Malaysia and Sumatra, Indonesia. Biochem Syst Ecol. 60:99–105.

[CIT0022] Tan MP, Jamsari AFJ, Siti Azizah MN 2012 Phylogeographic pattern of the striped snakehead, *Channa striata* in Sundaland: ancient river connectivity, geographical and anthropogenic singnatures. PLoS One. 7:e52089. 23284881 10.1371/journal.pone.0052089 PMC3527338

[CIT0023] Vidthayanon C 2012 Trichopodus pectoralis. The IUCN Red List of Threatened Species 2012: e.T188087A1852593. Gland (Switzerland): IUCN. 10.2305/IUCN.UK.2012-1.RLTS.T188087A1852593.en. 11 October 2018.

[CIT0024] Voris HK 2000 Maps of Pleistocene sea levels in Southeast Asia: shorelines, river systems and time durations. J Biogeography. 27:1153–1167.

[CIT0025] Ward RD 2012 FISH-BOL, a case study for DNA barcodes. DNA barcodes. Berlin (Germany): Springer; p. 423–439. 10.1007/978-1-61779-591-6_21 22684969

[CIT0029] Ward RD, Zemlak TS, Innes BH, Last PR, Hebert PDN 2005 DNA barcoding Australia’s fish species. Philos Trans Royal Soc B: Biol Sci. 360:1847–1857. doi: 10.1046/10.1098/rstb.2005.1716 PMC1609232 16214743

[CIT0026] Wares JP, Hughes AR, Grosberg RK. 2005 Mechanisms that drive evolutionary change. Insight from species introductions and invasions In: Sax DF, editor. Species invasions: insights into ecology, evolution and biogeography. Sunderland (MA): Sinauer Associates; p. 229–257.

[CIT0027] Welcomme RL 1988 International introductions of inland aquatic species. Rome (Italy): FAO Technical Paper: FAO; p. 294.

