# Supplementary material for: Hidden genetic diversity in snakeskin gourami, Trichopodus pectoralis (Perciformes, Osphronemidae), inferred from the mitochondrial DNA CO1 gene
Source: Mitochondrial DNA B Resour. 2019 Sep 12;4(2):2966–9. doi: 10.1080/23802359.2019.1662741 (PMC7707841; doi:10.1080/23802359.2019.1662741)
Supplement: Supplemental Material [file TMDN_A_1662741_SM3060.zip › Min Pau et al supplemental content.docx]

**Appendix**

**Table S1.** Sampling locality, coordinate and sample size (n) of *T. pectoralis* in this study

| Region | Population | | Latitude (North) | Longitude (East) | N |
| --- | --- | --- | --- | --- | --- |
| 1) East Peninsular Malaysia, Malaysia | 1) Jerantut, Pahang (JR) | | 3°56'14.62" | 102°21'43.34" | 5 |
|  | 2) Setiu, Terengganu (ST) | | 5° 4'25.81" | 103° 0'45.73" | 5 |
| 2) West Peninsular Malaysia, Malaysia | 3) Guar, Kedah (GR) | | 5°51'10.76" | 100°27'36.68" | 4 |
|  | 4) Lahat, Perak (LH) | | 4°32'27.15" | 101° 2'18.66" | 6 |
|  | 5) Sungai Besar, Selangor (SB) | | 3°40'31.43" | 100°59'8.84" | 2 |
| 3) Southern Peninsular Malaysia, Malaysia | 6) Muar, Johor (MR) | | 2°03'48.85" | 102°35'09.57" | 4 |
| 4) Malaysian Borneo, Malaysia | 7) Seri Aman, Sarawak (SA) | | 1°14'13.31" | 111°27'43.48" | 13 |
|  | 8) Serian, Sarawak (SR) | | 1°10'1.33" | 110°33'59.42" | 12 |
| 5) Vietnam | 9) Can Tho (CT) | | 9° 0'20.54" | 104°51'25.69" | 3 |
|  | 10) Bac Lieu (BL) | | 9°15'5.60" | 105°30'49.13" | 3 |
| 6) Thailand | 11) Lamphun (LP) | | 18°34'28.06" | 99° 0'31.40" | 5 |
|  | 12) Pattani (PT) | | 6°45'42.59" | 101°19'23.72" | 6 |
|  | 13) Ubon Ratchathani (UR) | | 15°11'4.83" | 104°52'12.47" | 16 |
| **Total** | | | | | **84** |
| 7) Indonesia | | GenBank accession number: KU692922 – 692927 | | | |
| 8) Philippines | | GenBank accession number: HQ682726 – 682730 | | | |
| 9) Myanmar | | GenBank accession number: LC190090 | | | |


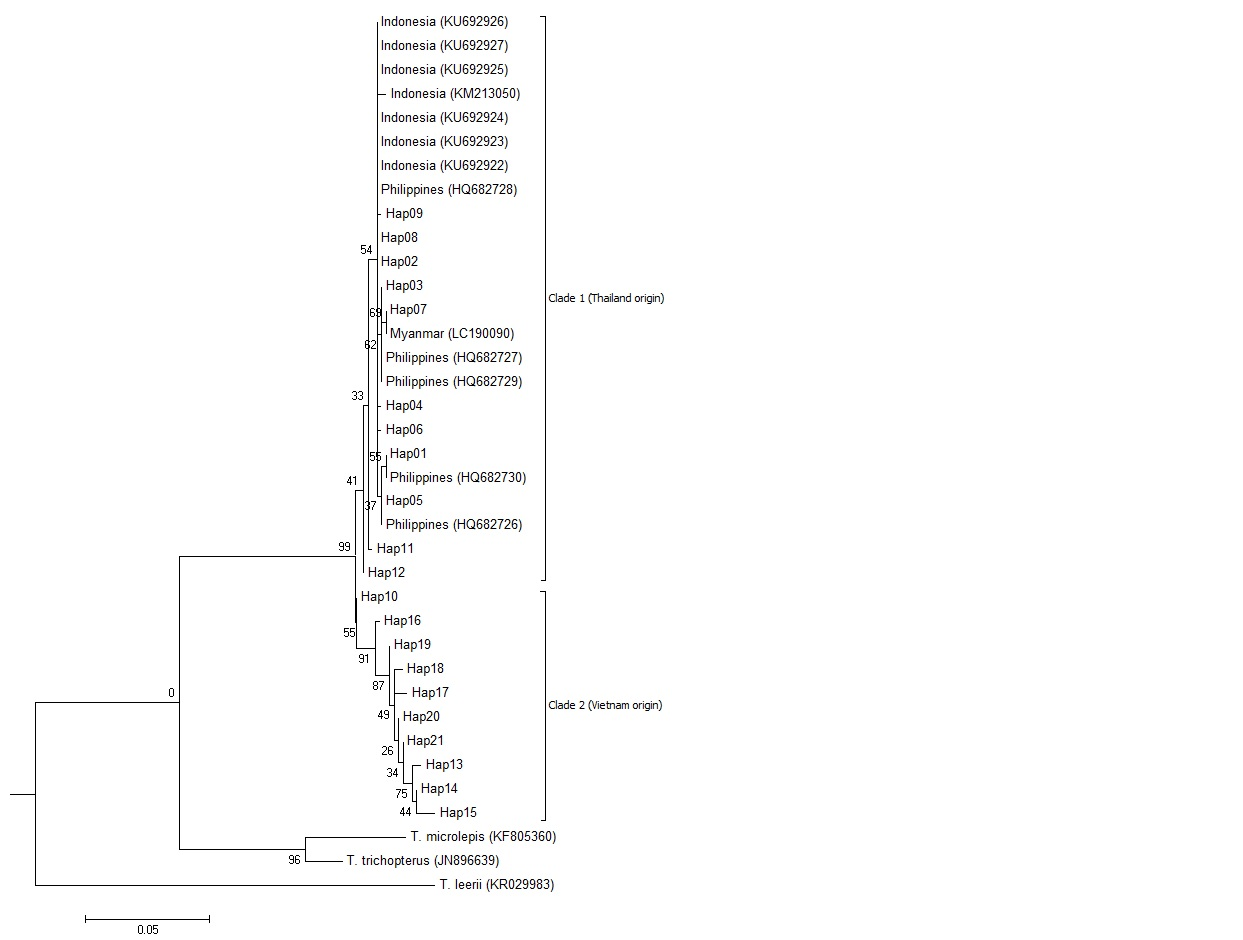


**Figure S1.** Evolutionary relationship of *T. pectoralis* haplotypes inferred from *coi* gene using ML method. Bootstrap value denoted at each branch.
